# Energy-Efficient Patching Strategy for Wireless Sensor Networks

**DOI:** 10.3390/s19020262

**Published:** 2019-01-10

**Authors:** Pengdeng Li, Lu-Xing Yang, Xiaofan Yang, Xiang Zhong, Junhao Wen, Qingyu Xiong

**Affiliations:** 1 School of Big Data & Software Engineering, Chongqing University, Chongqing 400044, China; pengdengli1992@cqu.edu.cn (P.L.); zx5587@hnu.edu.cn (X.Z.); jhwen@cqu.edu.cn (J.W.); xiong03@cqu.edu.cn (Q.X.); 2 School of Information Technology, Deakin University, Melbourne, VIC 3125, Australia; y.luxing@deakin.edu.au; 3 College of Mechanical and Vehicle Engineering, Hunan University, Changsha 410082, China

**Keywords:** wireless sensor network, computer virus, patching strategy, energy efficiency, optimal control theory, optimality system

## Abstract

Wireless sensor networks (WSNs) are vulnerable to computer viruses. To protect WSNs from virus attack, the virus library associated with each sensor node must be updated in a timely way. This article is devoted to developing energy-efficient patching strategies for WSNs. First, we model the original problem as an optimal control problem in which (a) each control stands for a patching strategy, and (b) the objective functional to be optimized stands for the energy efficiency of a patching strategy. Second, we prove that the optimal control problem is solvable. Next, we derive the optimality system for solving the optimal control problem, accompanied with a few examples. Finally, we examine the effects of some factors on the optimal control. The obtained results help improve the security of WSNs.

## 1. Introduction

**Figure 1:**
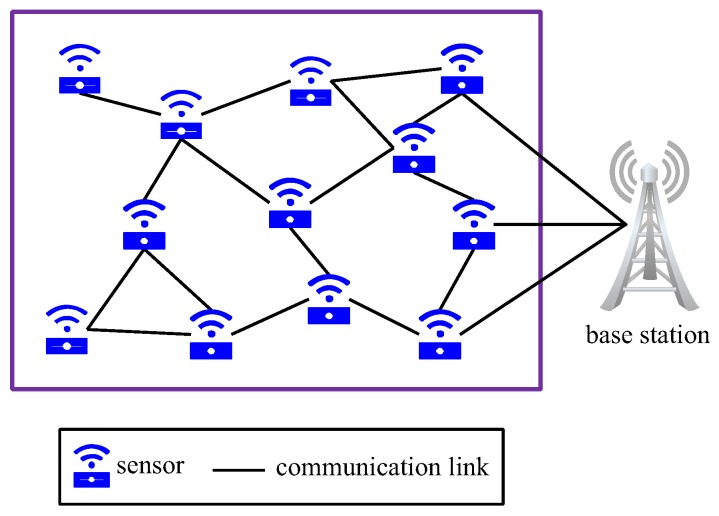
A small-sized WSN.

Smart sensor nodes, which are low-power devices equipped with a set of sensors, a processor, a memory, a power supply, a radio, and an actuator, can sense, measure, and gather information from the environment. Wireless sensor networks (WSNs), which are self-organized wireless networks of smart sensor nodes, are used to cooperatively transmit the sensed data to the base station [1]. See Figure 1 for a small-sized WSN. WSNs have important applications in many fields, ranging from military target surveillance and natural disaster relief to human health monitoring and hazardous environment exploration [2,3,4]. As WSNs are typically deployed in uncontrollable or even hostile environments, they are vulnerable to a wide range of cyberattacks. In particular, a cyber malefactor may launch a virus attack to the target WSN and perform intended malicious operations on each infected sensor node, ranging from stealing or falsifying the data in this node to destroying the node [5,6]. In the past few decades, numerous WSN-related virus accidents have been reported in the literature [7,8,9]. Consequently, protecting WSNs from virus attack has long been a major issue in the domain of cybersecurity [10].

### 1.1. The Energy-Efficient Patching Problem

**Figure 2:**
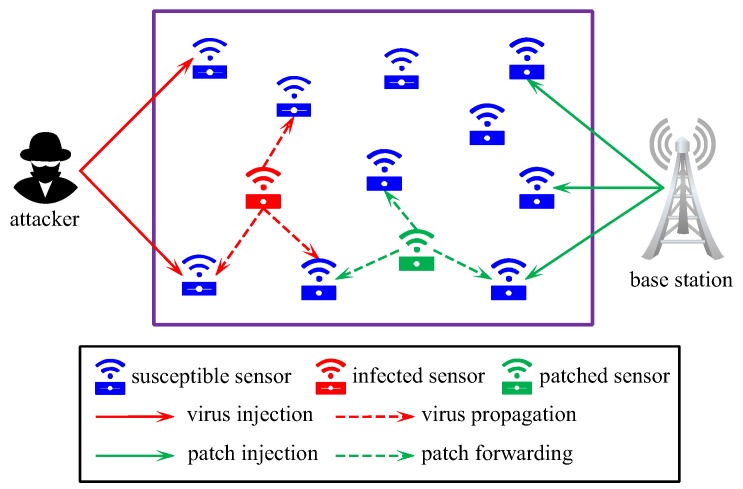
A diagram of patch injection and forwarding in the WSN shown in Figure 1.

To enable a WSN to automatically defend against virus attack, all sensor nodes in the network must be equipped with an intrusion response system (IRS). With the continual emergence of new viruses, the virus libraries associated with these IRSs must be updated in a timely manner to deal with new viruses [11]. For this purpose, new virus patches must be continually injected into a subset of sensor nodes from outside the network and then forwarded from patched node to unpatched node until the whole network is covered [12]. See Figure 2 for a diagram of patching the WSN shown in Figure 1. Technically, patching can be realized by reprogramming the underlying communication protocols [13,14,15]. From the perspective of defending against new viruses, new patches should be injected and forwarded as early as possible.

On the other hand, all sensor nodes in a WSN are with limited power resources. When the energy of a node is depleted, it will die and disconnect from the network [16,17,18]. As the lifetime of the network depends on the number of active nodes and the connectivity of the network, energy must be used efficiently to maximize the network lifetime. As patches are injected and forwarded at the energy cost, we face the following problem:

*Energy-efficient patching (EEP) problem: For a given WSN, develop an energy-efficient patching strategy.* To our knowledge, to date this problem has not been addressed. This paper focuses on the EEP problem.

### 1.2. Our Research Approach and Related Work

Optimal control theory deals with the problem of finding a control law for a given dynamic system such that a certain performance index is optimized [19,20]. Many practical problems have been resolved using this theory [21,22,23]. In this paper, we are going to deal with the EEP problem in the framework of optimal control theory. The key to accomplishing this task is to measure the energy efficiency of a patching strategy. For this purpose, we need to accurately characterize the propagating process of digital viruses over a WSN.

In recent years, several WSN-oriented virus propagation models have been suggested. All these models build on the premise that the sensor nodes are distributed uniformly over a highly regular region (rectangular or circular, say). As a result, the virus propagation process can be characterized by a coarse-grained compartmental epidemic model [24,25,26,27,28,29,30,31]. For most real-world WSNs, however, the sensor nodes are distributed nonuniformly over a highly irregular region. Unfortunately, none of the above models applies to such WSNs.

The fine-grained node-level epidemic modeling [32,33] is especially suited to the characterization of propagation processes on arbitrary networks, because in the modeling process the topological structure of the network rather than its geometrical shape is accounted for. In recent years, this modeling technique has been successfully applied to diverse areas such as epidemic spreading [34], malware spreading [35,36,37,38,39,40], rumor spreading [41,42], and cybersecurity [43,44,45]. In this article, we are going to employ this modeling technique to establish a WSN-oriented virus-patch mixed propagation model. On this basis, we will model the EEP problem as an optimal control problem.

### 1.3. Main Contributions

This paper is devoted to dealing with the EEP problem. Our main contributions are overviewed as follows.

- By employing the node-level epidemic modeling technique, we establish a WSN-based virus-patch mixed propagation model. Thereby, we measure the energy efficiency of a patching strategy. On this basis, we model the EEP problem as an optimal control problem we refer to as the *EEP model* in which (a) each control stands for a patching strategy, and (b) the objective functional to be optimized stands for the energy efficiency of a patching strategy.
- We show that the EEP model admits an optimal control and hence is solvable. We then give a necessary condition for optimal control of the EEP model, from which we conclude that the optimal control is bang-bang and hence is easily realizable. On this basis, we derive the optimality system for solving the EEP model and illustrate its application. Finally, we examine the effects of some factors on the optimal patching strategy.

The remaining materials are organized in this fashion: Section 2 introduces the EEP model. Section 3 and Section 4 present a method for solving the EEP model and give a few numeric examples, respectively. Section 5 reveals the effects of some factors on the optimal patching strategy. This work is closed by Section 6.

## 2. The Modeling of the Energy-Efficient Patching Problem

This section is devoted to the modeling of the EEP problem following these steps: (1) introduce basic terms and notations, (2) establish a WSN-related virus-patch mixed propagation model, (3) formulate a patching strategy, (4) measure the energy efficiency of a patching strategy, and (5) model the EEP problem as an optimal control problem.

### 2.1. Terms and Notations

Consider a WSN that operates in the time horizon [0,T]. Let V={1,2,⋯,N} denote the set of all sensor nodes in the network. Let G=(V,E) denote the topological structure of the network, i.e., {i,j}∈E if and only if nodes *i* and *j* are within the communication range of each other. Let $A={\left(a_{ij}\right)}_{N\times N}$ denote the adjacency matrix of *G*, i.e., $a_{ij}=1$ or 0 according as {i,j}∈E or not.

For our purpose, all nodes in the network are classified as three categories: *susceptible nodes*, *infected nodes*, and *patched nodes*. A susceptible node is one that is not infected with virus and has not received the newest patch. As a result, it is vulnerable to the viruses that can be handled only with the newest patch. An infected node is one that is infected with virus. A patched node is one that is not infected with virus and has received the newest patch. As a result, it is immune of all viruses. Let $X_{i}\left(t\right)=0$, 1, and 2 denote that node *i* is susceptible, infected, and patched at time *t*, respectively. Then the vector

(1) $$X\left(t\right)=\left(X_{1}\left(t\right),\cdots ,X_{N}\left(t\right)\right)$$

stands for the state of the network at time *t*. Let $S_{i}\left(t\right)$, $I_{i}\left(t\right)$, and $P_{i}\left(t\right)$ denote the probability of node *i* being susceptible, infected, and patched at time *t*, respectively.

(2) $$S_{i}\left(t\right)=\mathrm{Pr}\left\{X_{i}\left(t\right)=0\right\},\;I_{i}\left(t\right)=\mathrm{Pr}\left\{X_{i}\left(t\right)=1\right\},\;P_{i}\left(t\right)=\mathrm{Pr}\left\{X_{i}\left(t\right)=2\right\}.$$

As $S_{i}\left(t\right)=1-I_{i}\left(t\right)-P_{i}\left(t\right)$, the vector

(3) $$E\left(t\right)=\left(I_{1}\left(t\right),\cdots ,I_{N}\left(t\right),P_{1}\left(t\right),\cdots ,P_{N}\left(t\right)\right).$$

stands for the expected state of the network at time *t*.

**Remark** **1.** *The initial network expected state E(0) can be estimated employing network probe.*

### 2.2. A Virus-Patch Mixed Propagation Model

For our purpose, we need to establish a WSN-related virus-patch mixed propagation model. To this end, let us make a set of hypotheses as follows.

**Hypothesis** **1.** *Due to the emergence of new virus, each patched node becomes susceptible at an average rate of δ, which we refer to as the patch failure rate.*

**Hypothesis** **2.** *Due to the injection of a new virus, each susceptible node gets infected at an average rate of $\beta_{I}$. We refer to $\beta_{I}$ as the virus injection rate.*

**Hypothesis** **3.** *Due to the impact of the infected node j, each susceptible node i with $a_{ij}=1$ gets infected at an average rate of $\beta_{P}$, which we refer to as the virus propagation rate.*

**Hypothesis** **4.** *Due to the injection of new patch, each unpatched node gets patched at time t at the rate of $\gamma_{I}\left(t\right)$, which we refer to as the patch injection rate at time t.*

**Hypothesis** **5.** *Due to the influence of the patched node j, each unpatched node i with $a_{ij}=1$ gets patched at time t at the rate of $\gamma_{F}\left(t\right)$, which we refer to as the patch forwarding rate at time t.*

**Remark** **2.** *The patch failure rate δ, the virus injection rate $\beta_{I}$, and the virus propagation rate $\beta_{P}$ can be estimated through collecting and analyzing the relevant historical data.*

**Figure 3:**
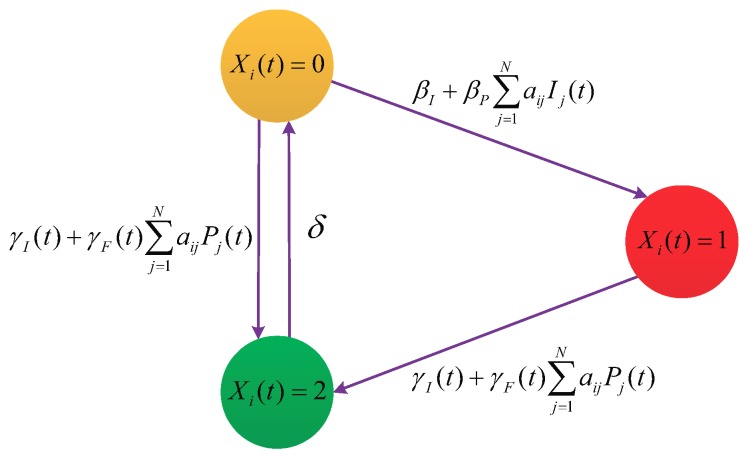
Diagram of the hypotheses (H${}_{1}$)–(H${}_{5}$).

Figure 3 shows the above hypotheses schematically. By the theory on continuous-time Markov chain [46], each susceptible node *i* gets infected at time *t* at an average rate of $\beta_{I}+\beta_{P}\sum_{j=1}^{N}a_{ij}I_{j}\left(t\right)$, and each infected node *i* gets patched at time *t* at the average rate of $\gamma_{I}\left(t\right)+\gamma_{F}\left(t\right)\sum_{j=1}^{N}a_{ij}P_{j}\left(t\right)$. It follows by Total Probability Formula that $I_{i}\left(t\right)$ ascends at time *t* at an average rate of

$$\left[\beta_{I}+\beta_{P}\sum_{j=1}^{N}a_{ij}I_{j}\left(t\right)\right]\left[1-I_{i}\left(t\right)-P_{i}\left(t\right)\right]-\left[\gamma_{I}\left(t\right)+\gamma_{F}\left(t\right)\sum_{j=1}^{N}a_{ij}P_{j}\left(t\right)\right]I_{i}\left(t\right).$$

Similarly, $P_{i}\left(t\right)$ ascends at time *t* at an average rate of

$$\left[\gamma_{I}\left(t\right)+\gamma_{F}\left(t\right)\sum_{j=1}^{N}a_{ij}P_{j}\left(t\right)\right]\left[1-P_{i}\left(t\right)\right]-\delta P_{i}\left(t\right).$$

Combining the above discussions, the expected state of the network obeys the following differential system:

(4) $$\left\{\begin{array}{cc} \frac{dI_{i}\left(t\right)}{dt} & =\left[\beta_{I}+\beta_{P}\sum_{j=1}^{N}a_{ij}I_{j}\left(t\right)\right]\left[1-I_{i}\left(t\right)-P_{i}\left(t\right)\right]-\left[\gamma_{I}\left(t\right)+\gamma_{F}\left(t\right)\sum_{j=1}^{N}a_{ij}P_{j}\left(t\right)\right]I_{i}\left(t\right),\\ \frac{dP_{i}\left(t\right)}{dt} & =\left[\gamma_{I}\left(t\right)+\gamma_{F}\left(t\right)\sum_{j=1}^{N}a_{ij}P_{j}\left(t\right)\right]\left[1-P_{i}\left(t\right)\right]-\delta P_{i}\left(t\right),\\ & \;0\leq t\leq T,1\leq i\leq N,\\ & \;E\left(0\right)=E_{0}. \end{array}\right.$$

We refer to the system as a *WSN-oriented virus-patch mixed propagation model*.

### 2.3. Formulating a Patching Strategy

We refer to the function $\gamma_{I}\left(t\right)$ (0≤t≤T) as a *patch injection strategy*, the function $\gamma_{F}\left(t\right)$ (0≤t≤T) as a *patch forwarding strategy*, and the two-dimensional vector-valued function u defined by

(5) $$u\left(t\right)=\left(\gamma_{I}\left(t\right),\gamma_{F}\left(t\right)\right),\;0\leq t\leq T,$$

as a *patching strategy*. The patching strategy is under control of the network administrator. In this paper, we assume the admissible set of patching strategy is

(6) $$U=\left\{u\in L{\left[0,T\right]}^{2}|\gamma_{I}\left(t\right)\leq \bar{\gamma_{I}},\gamma_{F}\left(t\right)\leq \bar{\gamma_{F}},0\leq t\leq T\right\},$$

where L[0,T] stands for the set of all Lebesgue integrable functions defined on the interval [0,T] [47].

**Remark** **3.** *The maximum allowable patch injection rate $\bar{\gamma_{I}}$ is determined by the budget for developing new patches. The maximum allowable patch forwarding rate $\bar{\gamma_{F}}$ is determined by the energy budget for forwarding patches.*

In this context, we may write the model (4) in matrix notation as follows.

(7) $$\left\{\begin{array}{cc} \frac{dE(t)}{dt} & =F(E(t),u(t)),\;0\leq t\leq T,\\ & E\left(0\right)=E_{0}. \end{array}\right.$$

### 2.4. Measuring the Energy Efficiency of a Patching Strategy

This subsection is dedicated to estimating the energy efficiency of a patching strategy. The energy efficiency of a patching strategy $u=(\gamma_{I},\gamma_{F})$ consists of two parts: the losses caused by viruses, and the energy cost used for patching. For our purpose, let us introduce a pair of hypotheses as follows.

**Hypothesis** **6.** *The average loss per unit time caused by each infected node is $w_{1}$ units (dollars, say), which we refer to as the loss coefficient.*

**Hypothesis** **7.** *The average energy cost per unit time used for each node to transmit or receive patches at a rate of γ is $w_{2}\gamma$ units. We refer to $w_{2}$ as the energy coefficient.*

**Remark** **4.** *The loss coefficient $w_{1}$ can be estimated by estimating the average value of the environmental data gained by the sensor nodes in the network. The energy coefficient $w_{2}$ is a common physical parameter of the sensor nodes in the network.*

According to the hypothesis (H${}_{6}$), the average loss caused by the node *i* in the infinitesimal time horizon [t,t+dt) is $w_{1}dt$ or zero according as this node is infected or not at time *t*. Therefore, the expected loss caused by the node *i* in the infinitesimal time horizon [t,t+dt) is $I_{i}\left(t\right)\cdot w_{1}dt+\left(1-I_{i}\left(t\right)\right)\cdot 0=w_{1}I_{i}\left(t\right)dt$. Hence, the expected loss of the whole network in the time horizon [0,T] is

(8) $$L\left(u\right)=w_{1}\int_{0}^{T}\sum_{i=1}^{N}I_{i}\left(t\right)dt.$$

Similarly, the expected energy overhead of the whole network for transmitting patches in the time horizon [0,T] is

(9) $$E_{T}\left(u\right)=w_{2}\int_{0}^{T}\gamma_{F}\left(t\right)\sum_{i=1}^{N}\left[1-P_{i}\left(t\right)\right]\sum_{j=1}^{N}a_{ij}P_{j}\left(t\right)dt.$$

and the expected energy overhead of the whole network for receiving patches in the time horizon [0,T] is

(10) $$E_{R}\left(u\right)=w_{2}\int_{0}^{T}\gamma_{I}\left(t\right)\sum_{i=1}^{N}\left[1-P_{i}\left(t\right)\right]dt+w_{2}\int_{0}^{T}\gamma_{F}\left(t\right)\sum_{i=1}^{N}\left[1-P_{i}\left(t\right)\right]\sum_{j=1}^{N}a_{ij}P_{j}\left(t\right)dt.$$

Hence, the expected energy overhead of the whole network for transmitting and receiving patches in the time horizon [0,T] is

(11) $$\begin{array}{cc} E(u) & =E_{T}\left(u\right)+E_{R}\left(u\right)\\ & =w_{2}\int_{0}^{T}\gamma_{I}\left(t\right)\sum_{i=1}^{N}\left[1-P_{i}\left(t\right)\right]dt+2w_{2}\int_{0}^{T}\gamma_{F}\left(t\right)\sum_{i=1}^{N}\left[1-P_{i}\left(t\right)\right]\sum_{j=1}^{N}a_{ij}P_{j}\left(t\right)dt. \end{array}$$

Combining the above discussions, we conclude that the energy efficiency of the patching strategy u can be measured by

(12) $$\begin{array}{cc} J(u) & =L(u)+E(u)\\ & =w_{1}\int_{0}^{T}\sum_{i=1}^{N}I_{i}\left(t\right)dt+w_{2}\int_{0}^{T}\gamma_{I}\left(t\right)\sum_{i=1}^{N}\left[1-P_{i}\left(t\right)\right]dt+2w_{2}\int_{0}^{T}\gamma_{F}\left(t\right)\sum_{i=1}^{N}\left[1-P_{i}\left(t\right)\right]\sum_{j=1}^{N}a_{ij}P_{j}\left(t\right)dt. \end{array}$$

### 2.5. The Modeling of the EEP Problem

Based on previous discussions, we model the EEP problem as the following optimal control problem:

(13) $$\begin{array}{c} {\mathrm{Min}}_{u\in U}J\left(u\right)=\int_{0}^{T}L\left(E\left(t\right),u\left(t\right)\right)dt\\ \mathrm{subject}\;\mathrm{to}\;\left\{\begin{array}{cc} \frac{dE(t)}{dt} & =F(E(t),u(t)),\;0\leq t\leq T,\\ & E\left(0\right)=E_{0}. \end{array}\right. \end{array}$$

Here,

(14) $$L\left(E\left(t\right),u\left(t\right)\right)=w_{1}\sum_{i=1}^{N}I_{i}\left(t\right)+w_{2}\gamma_{I}\left(t\right)\sum_{i=1}^{N}\left[1-P_{i}\left(t\right)\right]+2w_{2}\gamma_{F}\left(t\right)\sum_{i=1}^{N}\left[1-P_{i}\left(t\right)\right]\sum_{j=1}^{N}a_{ij}P_{j}\left(t\right).$$

We refer to this optimal control problem as the *EEP model*. In the model, each admissible control stands for an allowable patching strategy, the objective functional stands for the energy efficiency of an allowable patching strategy, and each optimal control stands for an EEP strategy.

The EEP model (13) is determined by the 10-tuple

(15) $$M=(G,T,\beta_{I},\beta_{P},\delta ,\bar{\gamma_{I}},\bar{\gamma_{P}},w_{1},w_{2},E_{0})$$

## 3. A Method for Solving the EEP Model

In the previous section, the EEP problem was modeled as an optimal control problem we refer to as the EEP model. This section is dedicated to deriving a systematic method for solving the EEP model using optimal control theory. First, we show that the EEP model is solvable. Second, we give a necessary condition for optimal control of the EEP model. On this basis, we present the optimality system for solving the EEP model.

### 3.1. The Solvability of the EEP Model

First, let us examine the solvability of the EEP model. For this purpose, we need the following lemma, which is a direct corollary of a classical theorem in optimal control theory [20].

**Lemma** **1.** *The EEP model (13) has an optimal control if the following five conditions hold simultaneously.*

*(C*_1_*)*  *U is closed and convex.*
*(C*_2_*)*  *There is u∈U such that the affiliated model (7) is solvable.*
*(C*_3_*)*  *F(E,u) is bounded by a linear function in E.*
*(C*_4_*)*  *L(E,u) is convex on U.*
*(C*_5_*)*  *$L\left(E,u\right)\geq c_{1}{∥u∥}_{2}^{\rho }+c_{2}$ for some ρ>1, $c_{1}>0$ and $c_{2}$.*

The main result in this subsection is given below.

**Theorem** **1.** *The EEP model (13) admits an optimal control.*

**Proof.**  Let $u=\left(\gamma_{I},\gamma_{F}\right)$ be a limit point of U. Then there is a sequence of points of U, $u^{\left(n\right)}=\left(\gamma_{I}^{\left(n\right)},\gamma_{F}^{\left(n\right)}\right),n=1,2,\cdots ,$ that approaches u. As the function space $L{\left[0,T\right]}^{2}$ is complete, we have $u\in L{\left[0,T\right]}^{2}$. Hence, the closeness of U follows from the observation that for 0≤t≤T, there hold

$$\gamma_{I}\left(t\right)=\lim_{n\to \infty }\gamma_{I}^{\left(n\right)}\left(t\right)\leq \bar{\gamma_{I}},\;\gamma_{F}\left(t\right)=\lim_{n\to \infty }\gamma_{F}^{\left(n\right)}\left(t\right)\leq \bar{\gamma_{F}}.$$

Let $u^{\left(1\right)},u^{\left(2\right)}\in U$, 0<α<1. As $L{\left[0,T\right]}^{2}$ is a real vector space, we have $\left(1-\alpha \right)u^{\left(1\right)}+\alpha u^{\left(2\right)}\in L{\left[0,T\right]}^{2}$. Hence, the convexity of U follows from the observation that for 0≤t≤T, there hold

$$\left(1-\alpha \right)\gamma_{I}^{\left(1\right)}\left(t\right)+\alpha \gamma_{I}^{\left(2\right)}\left(t\right)\leq \bar{\gamma_{I}},\;\left(1-\alpha \right)\gamma_{F}^{\left(1\right)}\left(t\right)+\alpha \gamma_{F}^{\left(2\right)}\left(t\right)\leq \bar{\gamma_{F}}.$$

The first condition of Lemma 1 is proven. Let $\bar{u}\left(t\right)\equiv \left(\bar{\gamma_{I}},\bar{\gamma_{F}}\right)$. Then $\bar{u}\in U$. As $F(E,\bar{u})$ is continuously differentiable, it follows by Continuation Theorem for Differential Systems [48] that the model (7) is solvable. The second condition is proven. The third condition follows from the boundedness of $I_{i}$, $P_{i}$, and u, and the fourth condition follows from that *L* is linear in u and hence is convex. The fifth condition follows from the observation that

$$L\left(E,u\right)\geq 0\geq \left(\gamma_{I}^{2}+\gamma_{F}^{2}\right)-\left({\bar{\gamma_{I}}}^{2}+{\bar{\gamma_{F}}}^{2}\right)={∥u∥}_{2}^{2}-\left({\bar{\gamma_{I}}}^{2}+{\bar{\gamma_{F}}}^{2}\right).$$

By Lemma 1, the proposition holds. □

### 3.2. A Necessary Condition for Optimal Control of the EEP Model

For our purpose, we need to give a necessary condition for optimal control of the EEP model. To this end, consider the Hamiltonian of the EEP model (13), which is given by

(16) $$\begin{array}{cc} H(E,u,z) & =w_{1}\sum_{i=1}^{N}I_{i}\left(t\right)+w_{2}\gamma_{I}\left(t\right)\sum_{i=1}^{N}\left[1-P_{i}\left(t\right)\right]+2w_{2}\gamma_{F}\left(t\right)\sum_{i=1}^{N}\left[1-P_{i}\left(t\right)\right]\sum_{j=1}^{N}a_{ij}P_{j}\left(t\right)\\ & \;+\sum_{i=1}^{N}\lambda_{i}\left(t\right)\left\{\left[\beta_{I}+\beta_{P}\sum_{j=1}^{N}a_{ij}I_{j}\left(t\right)\right]\left[1-I_{i}\left(t\right)-P_{i}\left(t\right)\right]-\left[\gamma_{I}\left(t\right)+\gamma_{F}\left(t\right)\sum_{j=1}^{N}a_{ij}P_{j}\left(t\right)\right]I_{i}\left(t\right)\right\}\\ & \;+\sum_{i=1}^{N}\mu_{i}\left(t\right)\left\{\left[\gamma_{I}\left(t\right)+\gamma_{F}\left(t\right)\sum_{j=1}^{N}a_{ij}P_{j}\left(t\right)\right]\left[1-P_{i}\left(t\right)\right]-\delta P_{i}\left(t\right)\right\}, \end{array}$$

where $z\left(t\right)=\left(\lambda_{1}\left(t\right),\cdots ,\lambda_{N}\left(t\right),\mu_{1}\left(t\right),\cdots ,\mu_{N}\left(t\right)\right)$ (0≤t≤T) is the adjoint.

A necessary condition for optimal control of the EEP model is given below.

**Theorem** **2.** *Suppose u is an optimal control of the EEP model (13), E is the solution to the affiliated model (7). Then there exists z with z(T)=0 such that*

(17) $$\left\{\begin{array}{cc} \frac{d\lambda_{i}\left(t\right)}{dt} & =-w_{1}+\lambda_{i}\left(t\right)\left[\beta_{I}+\beta_{P}\sum_{j=1}^{N}a_{ij}I_{j}\left(t\right)+\gamma_{I}\left(t\right)+\gamma_{F}\left(t\right)\sum_{j=1}^{N}a_{ij}P_{j}\left(t\right)\right]-\beta_{P}\sum_{j=1}^{N}a_{ji}\lambda_{j}\left(t\right)\left[1-I_{j}\left(t\right)-P_{j}\left(t\right)\right],\\ \frac{d\mu_{i}\left(t\right)}{dt} & =w_{2}\gamma_{I}\left(t\right)-\gamma_{F}\left(t\right)\sum_{j=1}^{N}a_{ji}\left[\left(2w_{2}+\mu_{j}\left(t\right)\right)\left(1-P_{j}\left(t\right)\right)-\lambda_{j}\left(t\right)I_{j}\left(t\right)\right]+2w_{2}\gamma_{F}\left(t\right)\sum_{j=1}^{N}a_{ij}P_{j}\left(t\right)\\ & \;+\lambda_{i}\left(t\right)\left[\beta_{I}+\beta_{P}\sum_{j=1}^{N}a_{ij}I_{j}\left(t\right)\right]+\mu_{i}\left(t\right)\left[\delta +\gamma_{I}\left(t\right)+\gamma_{F}\left(t\right)\sum_{j=1}^{N}a_{ij}P_{j}\left(t\right)\right],\\ & \;1\leq i\leq N,0\leq t\leq T. \end{array}\right.$$

*Moreover,*

(18) $$\gamma_{I}\left(t\right)=\left\{\begin{array}{c} 0\;if\;\sum_{i=1}^{N}\left(w_{2}+\mu_{i}\left(t\right)\right)\left(1-P_{i}\left(t\right)\right)>\sum_{i=1}^{N}\lambda_{i}\left(t\right)I_{i}\left(t\right),\\ \bar{\gamma_{I}}\;if\;\sum_{i=1}^{N}\left(w_{2}+\mu_{i}\left(t\right)\right)\left(1-P_{i}\left(t\right)\right)<\sum_{i=1}^{N}\lambda_{i}\left(t\right)I_{i}\left(t\right). \end{array}\right.$$

(19) $$\gamma_{F}\left(t\right)=\left\{\begin{array}{c} 0\;if\;\sum_{i=1}^{N}\left(2w_{2}+\mu_{i}\left(t\right)\right)\left(1-P_{i}\left(t\right)\right)\sum_{j=1}^{N}a_{ij}P_{j}\left(t\right)>\sum_{i=1}^{N}\lambda_{i}\left(t\right)I_{i}\left(t\right)\sum_{j=1}^{N}a_{ij}P_{j}\left(t\right),\\ \bar{\gamma_{F}}\;if\;\sum_{i=1}^{N}\left(2w_{2}+\mu_{i}\left(t\right)\right)\left(1-P_{i}\left(t\right)\right)\sum_{j=1}^{N}a_{ij}P_{j}\left(t\right)<\sum_{i=1}^{N}\lambda_{i}\left(t\right)I_{i}\left(t\right)\sum_{j=1}^{N}a_{ij}P_{j}\left(t\right). \end{array}\right.$$

**Proof.**  According to Pontryagin Minimum Principle [20], there exists z such that

$$\begin{array}{c} \frac{d\lambda_{i}\left(t\right)}{dt}=-\frac{\partial H(E(t),u(t),z(t))}{\partial I_{i}\left(t\right)},\;1\leq i\leq N,0\leq t\leq T,\\ \frac{d\mu_{i}\left(t\right)}{dt}=-\frac{\partial H(E(t),u(t),z(t))}{\partial P_{i}\left(t\right)},\;1\leq i\leq N,0\leq t\leq T. \end{array}$$

Thus, the system (17) follows by direct calculations. As the terminal cost is unspecified, and the final state is free, the transversality condition z(T)=0 holds. Finally, by using the optimality condition we have

$$u\left(t\right)=\arg\min_{\tilde{u}\in U}H\left(E\left(t\right),\tilde{u}\left(t\right),z\left(t\right)\right),\;0\leq t\leq T.$$

The systems (18) and (19) follow by direct calculations. □

**Remark** **5.** *By this theorem, every optimal control of the EEP model (13) is bang-bang and hence easily realizable.*

### 3.3. The Optimality System for the EEP Model

By optimal control theory, the systems (4), (17), (18), and (19) together with $E\left(0\right)=E_{0}$ and z(T)=0 constitute the optimality system for the EEP model (13). In view of the existence of optimal control, we can get an optimal control of the EEP model by solving the optimality system using Forward-Backward Euler Scheme.

## 4. Numerical Examples

**Figure 4:**
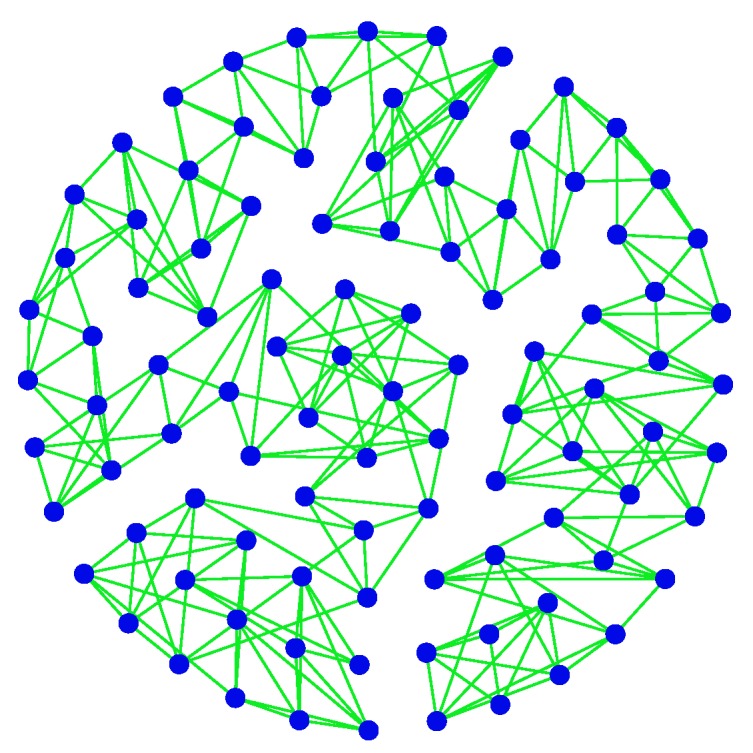
The subnet $G_{1}$ of *G*.

In the previous section, we presented the optimality system for solving the EEP model (13). In this section, we solve three instances of the EEP model to get their respective optimal controls. For this purpose, consider the real-world WSN given in [49] in which there are 66,917 nodes and 885,441 edges. Denote this network by *G*. First, we take a subnet $G_{1}$ with 100 nodes from *G*, which is plotted in Figure 4.

**Figure 5:**
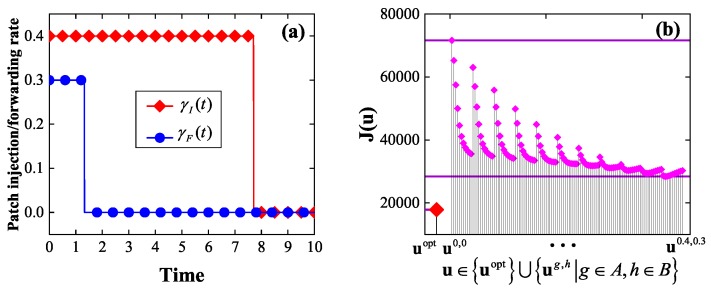
The experimental results in Example 1: (**a**) an optimal control, (**b**) a comparison between the optimal control and the set of static controls in terms of the energy efficiency.

**Example** **1.** *Consider the instance of the EEP model in which $G=G_{1}$, T=10, $\beta_{I}=0.2$, $\beta_{P}=0.15$, δ=0.1, $w_{1}=w_{2}=1$, $\bar{\gamma_{I}}=0.4$, $\bar{\gamma_{F}}=0.3$, and $E_{0}=\left(0.3,\cdots ,0.3\right)$. By solving the corresponding optimality system, we get an optimal control $u^{opt}$, which is shown in Figure 5a. It is seen that either of the two components of $u^{opt}$ is bang-bang, as expected by Theorem 2. Furthermore, it is seen that either of the two components of $u^{opt}$ first stays at the maximum allowable rate, then abruptly drops to the zero rate, and finally stays at the zero rate.*

*Let A={0,0.04,0.08,⋯,0.4}, B={0,0.03,0.06,⋯,0.3}. For g∈A, h∈B, let $u^{g,h}=\left(\gamma_{I}^{g,h},\gamma_{F}^{g,h}\right)$ denote the static control with $\gamma_{I}^{g,h}\left(t\right)=g$, $\gamma_{F}^{g,h}\left(t\right)=h$, 0≤t≤T. For comparative purpose, Figure 5b plots J(u) for all $u\in \left\{u^{opt}\right\}⋃\left\{u^{g,h}|g\in A,h\in B\right\}$. It is seen that $u^{opt}$ is superior to all the static controls in terms of the energy efficiency, as expected.*

**Figure 6:**
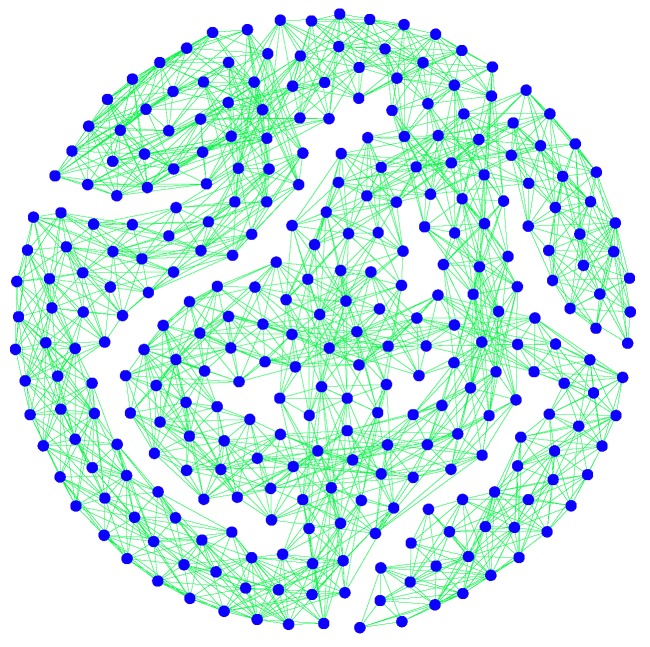
The subnet $G_{2}$ of *G*.

Second, we take a subnet $G_{2}$ with 300 nodes from *G*, which is exhibited in Figure 6.

**Figure 7:**
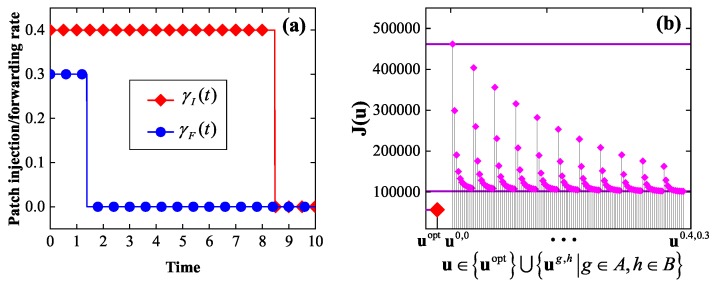
The experimental results in Example 2: (**a**) an optimal control, (**b**) a comparison between the optimal control and the set of static controls in terms of the energy efficiency.

**Example** **2.** *Consider the instance of the EEP model in which $G=G_{2}$, T=10, $\beta_{I}=0.2$, $\beta_{P}=0.15$, δ=0.1, $w_{1}=w_{2}=1$, $\bar{\gamma_{I}}=0.4$, $\bar{\gamma_{F}}=0.3$, and $E_{0}=\left(0.3,\cdots ,0.3\right)$. By solving the corresponding optimality system, we get an optimal control $u^{opt}$, which is shown in Figure 7a. It is seen that either of the two components of $u^{opt}$ is bang-bang, as expected by Theorem 2. Again, it is seen that either of the two components of $u^{opt}$ first stays at the maximum allowable rate, then abruptly drops to the zero rate, and finally stays at the zero rate.*

*Let A={0,0.04,0.08,⋯,0.4}, B={0,0.03,0.06,⋯,0.3}. For g∈A, h∈B, let $u^{g,h}=\left(\gamma_{I}^{g,h},\gamma_{F}^{g,h}\right)$ denote the static control with $\gamma_{I}^{g,h}\left(t\right)=g$, $\gamma_{F}^{g,h}\left(t\right)=h$, 0≤t≤T. For comparative purpose, Figure 7b plots J(u) for all $u\in \left\{u^{opt}\right\}⋃\left\{u^{g,h}|g\in A,h\in B\right\}$. Again, it is seen that $u^{opt}$ outperforms all the static controls in terms of the energy efficiency, as expected.*

**Figure 8:**
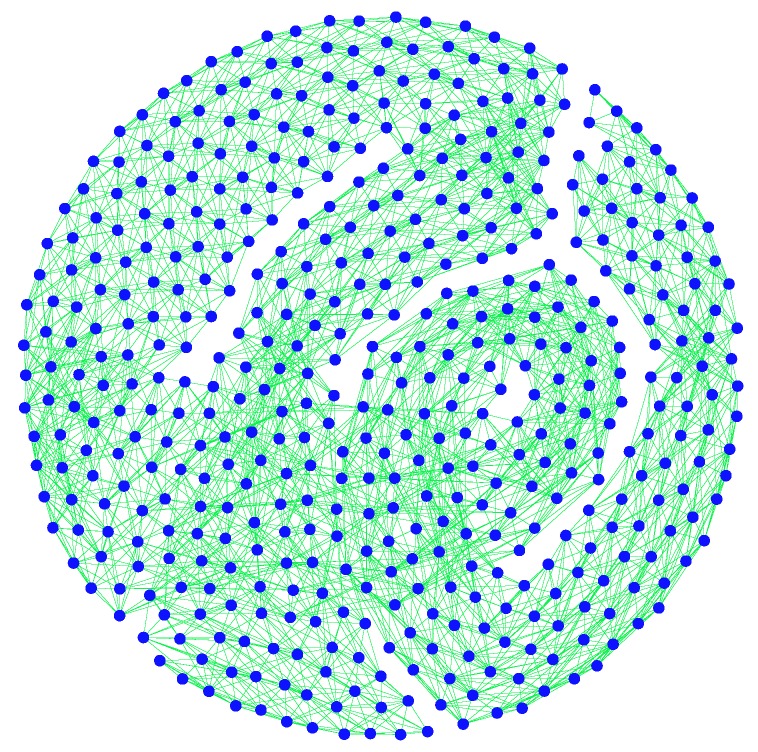
The subnet $G_{3}$ of *G*.

Finally, we take a subnet $G_{3}$ with 500 nodes from *G*, which is displayed in Figure 8.

**Figure 9:**
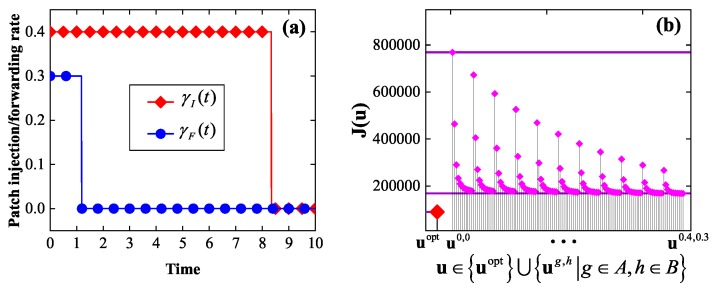
The experimental results in Example 3: (**a**) an optimal control, (**b**) a comparison between the optimal control and the set of static controls in terms of the energy efficiency.

**Example** **3.** *Consider the instance of the EEP model in which $G=G_{3}$, T=10, $\beta_{I}=0.2$, $\beta_{P}=0.15$, δ=0.1, $w_{1}=w_{2}=1$, $\bar{\gamma_{I}}=0.4$, $\bar{\gamma_{F}}=0.3$, and $E_{0}=\left(0.3,\cdots ,0.3\right)$. By solving the corresponding optimality system, we get an optimal control $u^{opt}$, which is shown in Figure 9a. It is seen that either of the two components of $u^{opt}$ is bang-bang, as expected by Theorem 2. Once more, it is seen that either of the two components of $u^{opt}$ first stays at the maximum allowable rate, then abruptly drops to the zero rate, and finally stays at the zero rate.*

*Let A={0,0.04,0.08,⋯,0.4}, B={0,0.03,0.06,⋯,0.3}. For g∈A, h∈B, let $u^{g,h}=\left(\gamma_{I}^{g,h},\gamma_{F}^{g,h}\right)$ denote the static control with $\gamma_{I}^{g,h}\left(t\right)=g$, $\gamma_{F}^{g,h}\left(t\right)=h$, 0≤t≤T. For comparative purpose, Figure 9b plots J(u) for all $u\in \left\{u^{opt}\right\}⋃\left\{u^{g,h}|g\in A,h\in B\right\}$. Also, it is seen that $u^{opt}$ overmatches all the static controls in terms of the energy efficiency, as expected.*

From the above three examples and 100 similar examples, we conclude the following results:

(i) For each instance of the EEP model, the patch injection strategy in the optimal patching strategy obtained by solving the optimality system first attains the maximum allowable patch injection rate for a period of time, then jumps sharply to the zero rate, and finally keeps the zero rate for the remaining period of time.
(ii) For each instance of the EEP model, the patch forwarding strategy in the optimal patching strategy obtained by solving the optimality system first attains the maximum allowable patch forwarding rate for a period of time, then jumps sharply to the zero rate, and finally keeps the zero rate for the remaining period of time.

In practice, such patching strategies are easily implementable. Therefore, we recommend to WSN administrators the EEP strategies obtained in this way.

## 5. Further Discussions

In the previous section, a method for calculating EEP strategies for WSNs was presented. In this section, we experimentally examine the effects of some factors on the optimal patching strategy obtained in this way.

### 5.1. The Effects of the Loss and Energy Coefficients

First, we study the effects of the loss coefficient and the energy coefficient on the optimal patching strategy, respectively.

**Experiment** **1.** *Consider a set of instances of the EEP model in which $G\in \{G_{1},G_{2},G_{3}\}$, T=10, $\beta_{I}=0.1$, $\beta_{P}=0.2$, δ=0.1, $\bar{\gamma_{I}}=0.4$, $\bar{\gamma_{F}}=0.3$, and E(0)=(0.3,⋯,0.3).*

**Figure 10:**
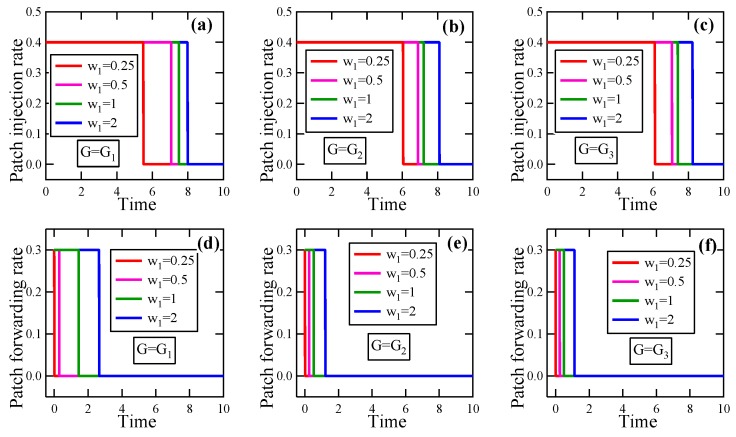
The experimental results in Experiment 1 about the effect of the loss coefficient on the optimal patching strategy. (**a**) Patch injection rate, $G=G_{1}$; (**b**) Patch injection rate, $G=G_{2}$; (**c**) Patch injection rate, $G=G_{3}$; (**d**) Patch forwarding rate, $G=G_{1}$; (**e**) Patch forwarding rate, $G=G_{2}$; (**f**) Patch forwarding rate, $G=G_{3}$.

**Figure 11:**
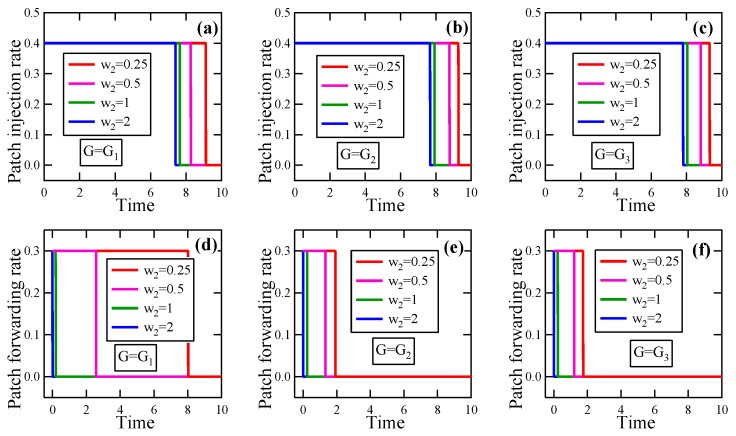
The experimental results in Experiment 1 about the effect of the energy coefficient on the optimal patching strategy. (**a**) Patch injection rate, $G=G_{1}$; (**b**) Patch injection rate, $G=G_{2}$; (**c**) Patch injection rate, $G=G_{3}$; (**d**) Patch forwarding rate, $G=G_{1}$; (**e**) Patch forwarding rate, $G=G_{2}$; (**f**) Patch forwarding rate, $G=G_{3}$.

*(a)*  *Suppose $w_{2}=1$, $w_{1}\in \left\{0.25,0.5,1,2\right\}$. By solving these optimality systems, we get the respective optimal patching strategies, in which the patch injection strategies and the patch forwarding strategies are depicted in Figure 10a–c and Figure 10c–f, respectively. It is seen that with the increase of $w_{1}$, the jump point of either of the patch injection strategy and the patch forwarding strategy in $u^{opt}$ moves to the right.*
*(b)*  *Suppose $w_{1}=1$, $w_{2}\in \left\{0.25,0.5,1,2\right\}$. By solving these optimality systems, we get the respective optimal patching strategies, in which the patch injection strategies and the patch forwarding strategies are exhibited in Figure 11a–c and Figure 11c–f, respectively. It is seen that with the increase of $w_{2}$, the jump point of either of the patch injection strategy and the patch forwarding strategy in $u^{opt}$ moves to the left.*

From this experiment and 100 similar experiments, we conclude the following results about the EEP model:

(i) With the increase of the loss coefficient, the jump point of the patch injection strategy in the optimal patching strategy obtained by solving the optimality system moves to the right, as does the jump point of the patch forwarding strategy in the optimal patching strategy. Hence, with the increase of the loss coefficient, the energy overhead for patching must be enhanced to achieve a higher energy efficiency.
(ii) With the increase of the energy coefficient, the jump point of the patch injection strategy in the optimal patching strategy obtained by solving the optimality system moves to the left, as does the jump point of the patch forwarding strategy in the optimal patching strategy. Hence, with the increase of the energy coefficient, the energy overhead for patching must be reduced to achieve a higher energy efficiency.

### 5.2. The Effects of the Maximum Allowable Patch Injection and Forwarding Rates

Second, let us inspect the effects of the maximum allowable patch injection rate and the maximum allowable forwarding rates on the optimal patching strategy, respectively.

**Experiment** **2.** *Consider a set of instances of the EEP model in which $G\in \{G_{1},G_{2},G_{3}\}$, T=10, $\beta_{I}=0.1$, $\beta_{P}=0.2$, δ=0.15, $w_{1}=w_{2}=1$, and $E_{0}=\left(0.3,\cdots ,0.3\right)$.*

**Figure 12:**
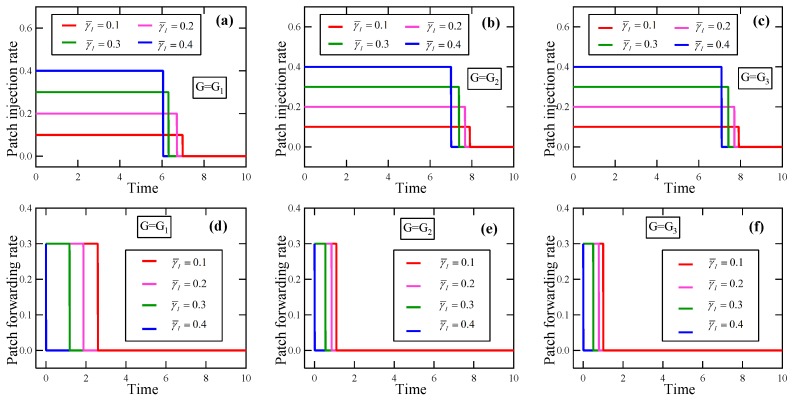
The experimental results in Experiment 2 about the effect of the maximum allowable patch injection rate on the optimal patching strategy. (**a**) Patch injection rate, $G=G_{1}$; (**b**) Patch injection rate, $G=G_{2}$; (**c**) Patch injection rate, $G=G_{3}$; (**d**) Patch forwarding rate, $G=G_{1}$; (**e**) Patch forwarding rate, $G=G_{2}$; (**f**) Patch forwarding rate, $G=G_{3}$.

**Figure 13:**
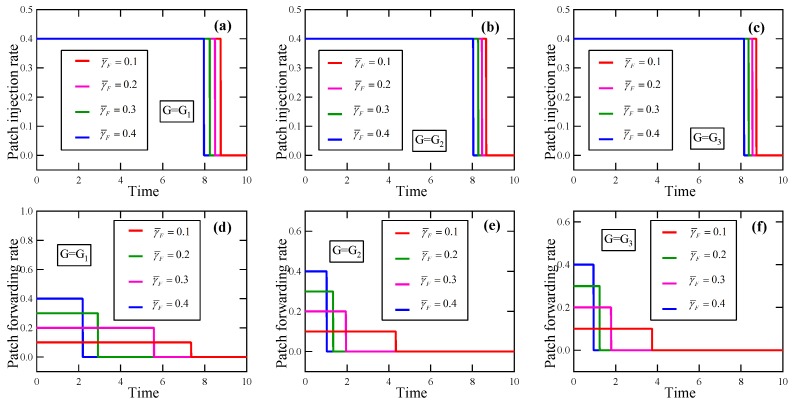
The experimental results in Experiment 2 about the effect of the maximum allowable patch forwarding rate on the optimal patching strategy. (**a**) Patch injection rate, $G=G_{1}$; (**b**) Patch injection rate, $G=G_{2}$; (**c**) Patch injection rate, $G=G_{3}$; (**d**) Patch forwarding rate, $G=G_{1}$; (**e**) Patch forwarding rate, $G=G_{2}$; (**f**) Patch forwarding rate, $G=G_{3}$.

*(a)*  *Suppose $\bar{\gamma_{F}}=0.3$, $\bar{\gamma_{I}}\in \left\{0.1,0.2,0.3,0.4\right\}$. By solving these optimality systems, we get the respective optimal patching strategies, in which the patch injection strategies are plotted in Figure 12a–c, and the patch forwarding strategies are portrayed in Figure 12d–f. It is seen that with the increase of $\bar{\gamma_{I}}$, the jump point of the patch injection strategy in $u^{opt}$ moves to the left, so does the jump point of the patch forwarding strategy in $u^{opt}$.*
*(b)*  *Suppose $\bar{\gamma_{I}}=0.3$, $\bar{\gamma_{F}}\in \left\{0.1,0.2,0.3,0.4\right\}$. By solving these optimality systems, we get the respective optimal patching strategies, in which the patch injection strategies are displayed in Figure 13a–c, and the patch forwarding strategies are depicted in Figure 13d–f. It is seen that with the increase of $\bar{\gamma_{F}}$, the jump point of the patch injection strategy in $u^{opt}$ moves to the left, so does the jump point of the patch forwarding strategy in $u^{opt}$.*

From this experiment and 100 similar expects, we conclude the following results about the EEP problem:

(i) With the increase of the maximum allowable patch injection rate, the jump point of the patch injection strategy in the optimal patching strategy moves to the left, so does the jump point of the patch forwarding strategy in the optimal patching strategy. Hence, with the increase of the maximum allowable patch injection rate, the energy overhead for patching must be reduced to achieve a higher energy efficiency.
(ii) With the increase of the maximum allowable patch forwarding rate, the jump point of the patch injection strategy in the optimal patching strategy moves to the left, so does the jump point of the patch forwarding strategy in the optimal patching strategy. Hence, with the increase of the maximum allowable patch forwarding rate, the energy overhead for patching must be reduced to achieve a higher energy efficiency.

## 6. Concluding Remarks

This article has studied the problem of developing EEP strategies for WSNs. Based on a novel virus-patch mixed propagation model, the problem has been modeled as an optimal control problem. The solvability of this optimal control problem has been proved, and a systematic method for solving the optimal control problem has been presented. These results may help us to defending against virus attacks to WSNs in an energy-efficient way.

Still, there are some relevant problems that are worth study. The practicality of the proposed EEP strategies should be considered very carefully. This work builds on the premise that virus patches can be injected into any of the sensor nodes in the WSN. In practice, however, patches can be injected into only those nodes that are in the proximity of the base station. Therefore, this work should be extended to such scenarios. In this paper, the virus attack strategy is assumed to be static. In practice, the malefactor may intelligently change the attack strategy over time to gain a larger benefit. In this situation, it is appropriate to study the EEP problem in the framework of game theory [50,51,52,53,54,55,56].

## Abbreviations

The following abbreviations are used in this manuscript:

WSN	Wireless sensor network
IRS	Intrusion response system
EEP	Energy-efficient patching
